# Neurological and neurosurgical conditions presenting first with ophthalmological clinical features: A case series

**DOI:** 10.22336/rjo.2025.41

**Published:** 2025

**Authors:** Anchal Tripathi, Ashish Pandey, Radhika Gupta, Amul Gupta, Nitin Vichare, Faiz Ahmad

**Affiliations:** 1 Department of Ophthalmology, Military Hospital, Jammu, Jammu and Kashmir, India; 2 Department of Radiology, Military Hospital, Jammu, Jammu and Kashmir, India; 3 Department of Neurology, Military Hospital, Jammu, Jammu and Kashmir, India

**Keywords:** neurological diseases in the eye, intracranial aneurysm, multiple myeloma, Miller Fisher syndrome, Neuromyelitis optica, CT = Computed Tomography, ICA = Internal Carotid Artery, FLAIR = Fluid-Attenuated Inversion Recovery, STIR = Short Tau Inversion Recovery

## Abstract

**Objective:**

We report four unique neurological cases that initially presented with ophthalmological manifestations.

**Case series:**

In the first case, a 36-year-old man came in with a left-sided headache and isolated third nerve palsy. Detailed investigations led to the discovery of a supraclinoid internal carotid artery aneurysm, necessitating prompt neurosurgical intervention. The second case featured a 74-year-old man with complete drooping of his right eyelid. The diagnosis of complete third nerve palsy led to further tests, which uncovered multiple myeloma. The third case involved a 19-year-old girl who had persistent double vision and bilateral ptosis. A collaborative effort between ophthalmologists and neurologists revealed Miller Fisher syndrome, which was successfully treated with intravenous immunoglobulins. Lastly, the fourth case described an 8-year-old girl who presented with optic neuritis. A thorough ophthalmological evaluation led to the diagnosis of Neuromyelitis Optica Spectrum Disorder, enabling early treatment and significant improvement.

**Discussion:**

These cases highlight the crucial role of ophthalmologists in identifying severe systemic and neurological conditions through subtle ocular signs. Each diagnosis—from aneurysms to autoimmune and malignant disorders—was made possible by vigilant ophthalmic evaluation and timely interdisciplinary collaboration. These examples reinforce that the eye often provides the first clue to an underlying disease, and that early recognition by ophthalmologists can be critical to effective, and sometimes lifesaving, intervention.

**Conclusion:**

These cases collectively emphasize the critical importance of detailed ocular evaluation in the early detection and management of a wide range of neurological and systemic conditions, highlighting how ocular symptoms can often be the first indication of more serious underlying health issues.

## Introduction

The eyes can serve as a window into a patient’s overall health, providing crucial insights into broader neurological or systemic issues. This case series illustrates how comprehensive ocular evaluations can reveal hidden pathologies, from unruptured intracranial aneurysms to multiple myeloma (MM) and neuromyelitis optica spectrum disorders (NMOSD).

Unruptured intracranial aneurysms, commonly seen at the bifurcation of arteries in the circle of Willis, are typically asymptomatic until they rupture, leading to a life-threatening subarachnoid hemorrhage [1,2]. Similarly, small internal carotid artery aneurysms may cause a local mass effect as they grow, potentially compressing adjacent structures and causing symptoms such as cranial nerve palsies or visual disturbances [3-5].

Multiple myeloma involving the central nervous system (CNS) has a variety of presentations, often causing bone lesions and significant pain [6,7]. Localized plasmacytomas can also arise from the cranium, dura, meninges, or brain parenchyma, complicating diagnosis [8-10]. Clinical symptoms such as extremity weakness, altered mental status, gait disturbances, or cranial nerve palsies can be challenging to diagnose due to their varied causes [9-11].

Miller Fisher syndrome (MFS), a variant of Guillain-Barré syndrome (GBS), is typically characterized by ophthalmoplegia, ataxia, and areflexia [12,13]. However, incomplete forms of MFS may present with only one or two of these clinical findings [13-15]. A respiratory or gastrointestinal tract illness often precedes this acute polyneuropathy and may be associated with antibodies, such as anti-GQ1b, further complicating the diagnostic landscape [14-17].

NMOSD and anti-myelin oligodendrocyte glycoprotein (anti-MOG) syndromes, which are inflammatory conditions of the central nervous system (CNS), often involve the optic nerves and the spinal cord [18,19]. Their clinical manifestations and habitual relapsing course can be confused with multiple sclerosis (MS), making accurate diagnosis critical, especially since some MS treatments can be detrimental to patients with NMOSD [19-22]. Studies have shown that various immune cells, such as neutrophils, macrophages, and eosinophils, play significant roles in the formation of NMOSD lesions, contributing to tissue damage through diverse mechanisms [19-22].

This case series examines three distinct cases where patients initially presented to the Ophthalmology outpatient department, highlighting the crucial role of comprehensive ocular evaluations in identifying underlying neurological diseases.

## Case Series

### Case 1: Supraclinoid internal carotid artery aneurysm

A 36-year-old male presented to the Ophthalmology outpatient department complaining of a severe headache for two weeks. The headache was unilateral, affecting the left side, and was described as throbbing in nature. Along with the headache, the patient experienced a few episodes of nausea and vomiting. Although the headache partially responded to over-the-counter medications such as paracetamol and naproxen, the symptoms did not fully subside, leading him to seek further medical advice.

**Fig. 1:**
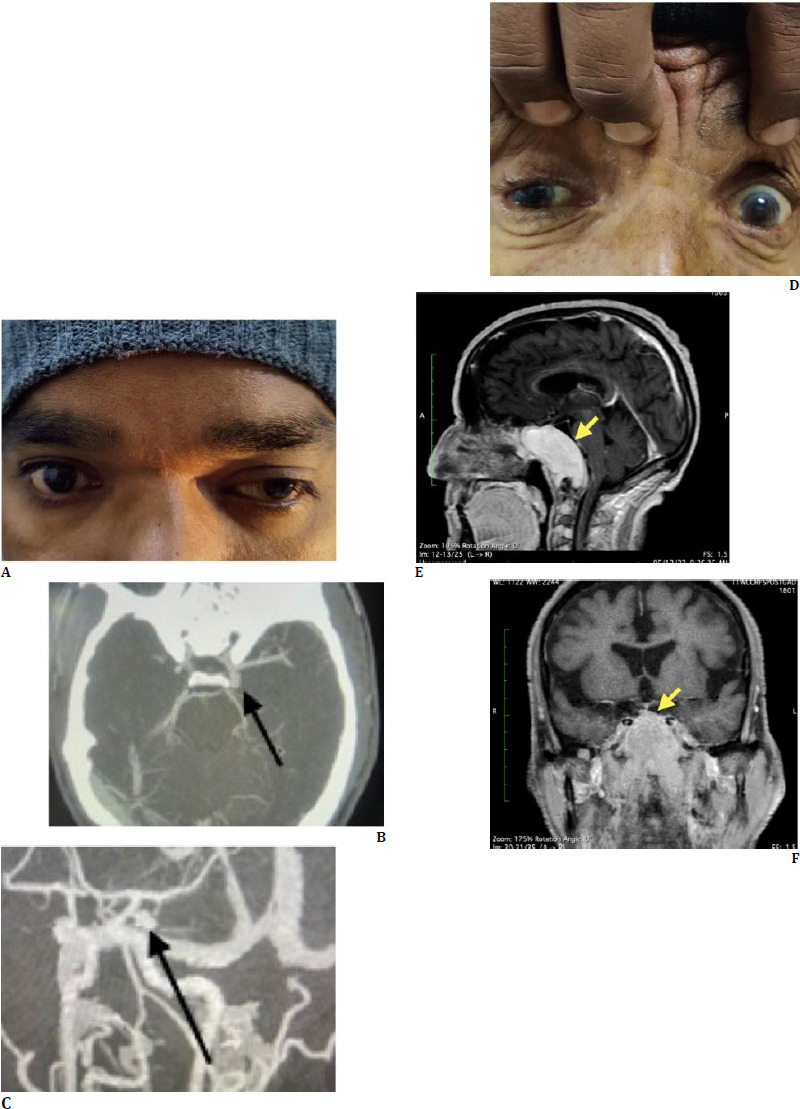
**A**. Left eye of a young male shows mild ptosis with down and out appearance in primary gaze; **B and C** CT Angiography reveals bilobed aneurysm (black arrow) arising from the terminal left ICA that is directed posteriorly; **D**. Right eye of an elderly male, presenting with complete ptosis (image not shown), shows down and out appearance in primary gaze; **E**. Post contrast T1 sagittal section shows intense homogenous enhancement of a lobulated mass epicentered in the clivus; **F**. Coronal T1 image shows intense homogenous enhancement that is involving bilateral cavernous sinuses. The lesion is also encasing the bilateral internal carotid arteries (ICAs). A few foci of blooming are also visible (images not shown) in the lesion

During the ophthalmological examination, the best corrected visual acuity (BCVA) was found to be 6/6 in both eyes. The patient was found to have an isolated partial third nerve palsy of the left side, characterized by mild ptosis and ocular muscle weakness (**Fig. 1A**). No other significant past medical or surgical history was noted. The patient denied any history of trauma, fever, seizures, motor or sensory deficits, or similar previous episodes.

Suspecting a potential neurological cause, a magnetic resonance imaging (MRI) of the brain and orbit was ordered, which was reported as usual. However, due to the sudden onset of the symptoms and high index of suspicion, computed tomography (CT) angiography was conducted, revealing a saccular aneurysm in the left supraclinoid segment of the internal carotid artery (ICA) (**Fig. 1B, C**). This finding was significant, as unruptured intracranial aneurysms, especially in this location, can lead to life-threatening subarachnoid hemorrhage if they rupture.

Given the critical nature of the diagnosis and the risk of rupture, the patient was promptly referred for neurosurgical intervention, where he underwent endovascular surgery and recovered well.

The successful identification and rapid neurosurgical response ultimately ensured the patient’s safety and well-being.

### Case 2: Multiple Myeloma

A 74-year-old man presented to the Ophthalmology outpatient department with complaints of complete drooping of the right eyelid. He had consulted various doctors previously, but his symptoms persisted. The patient had no significant past medical or surgical history, and his vital signs were within normal limits. On ophthalmological evaluation, his best-corrected visual acuity (BCVA) was found to be 6/6 bilaterally. He exhibited severe ptosis, with limitation of adduction, depression, and elevation in the right eye, suggesting dysfunction of the oculomotor nerve (Fig. 1D). The pupils were equal in size, round, and reactive to light, with no relative afferent pupillary defect (RAPD). Fundoscopy revealed bilateral normal findings.

Given the clinical findings, an urgent contrast-enhanced MRI (CEMRI) was requested, which revealed a T2 iso-intense, lobulated mass centered in the clivus with central hypo-intensity on the T2 axial image. On post-contrast T1 sagittal and coronal images, the lesion showed intense homogenous enhancement and involved bilateral cavernous sinuses (**Fig. 1E, F**). The lesion also encased the bilateral internal carotid arteries (ICAs). A few foci of blooming were also observed in the lesion. These findings suggested a likely malignancy, prompting further hematological investigations. Blood tests revealed an accelerated erythrocyte sedimentation rate (ESR) of 125 mm/h, along with severe normocytic normochromic anemia and rouleaux formation. A detailed examination of the patient’s blood proteins revealed elevated total protein levels (11.57 g/dL), as well as elevated immunoglobulin A (IgA) (6.15 g/dL) and immunoglobulin G (IgG) (4.01 g/dL), with an M-spike. The presence of Bence-Jones proteins in the urine analysis further supported the suspicion of multiple myeloma.

The patient was then referred to an oncologist for further management. There, the patient was diagnosed with multiple myeloma based on bone marrow biopsy findings, and the treatment was promptly initiated on the oncology side.

### Case 3: Miller Fisher syndrome (MFS)

**Fig. 2:**
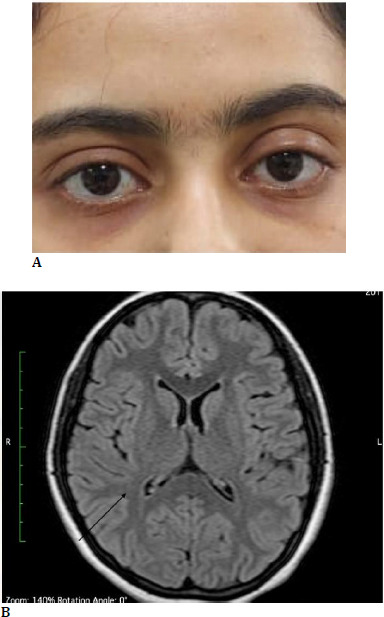
**A**. Bilateral mild ptosis with restriction of ocular movements in a young female; **B**. Axial FLAIR image - no abnormality is observed

A 19-year-old girl visited the Ophthalmology outpatient department seeking treatment for persistent diplopia. Despite prior treatment attempts at nearby hospitals, her condition remained unresolved, leading her to seek further medical advice. During the ophthalmological evaluation, the patient exhibited bilateral ptosis with significant limitation in ocular movements (**Fig. 2A**), indicating a potential neurological cause. Orthoptic examination revealed esotropia of 8 prism diopters (PD) at the near distance and 18 PD at the far distance, suggesting an imbalance in eye muscle control.

The patient’s history revealed no recent illness or known trauma, and she denied experiencing worsening double vision with specific eye movements. Her ptosis displayed no diurnal variation, which might otherwise suggest conditions like myasthenia gravis. The Humphrey Kinetic Visual Field test and fundus examination yielded normal results, with no signs of abnormality in the optic nerve head or retinal pathology. The pupils were round, equal in size, and reactive to light, with no RAPD. However, the noticeable ptosis and ophthalmoplegia raised suspicions of an underlying neurological condition.

The laboratory tests, including a complete blood count, erythrocyte sedimentation rate, C-reactive protein, electrolytes, and urine analysis, showed no significant abnormalities. Renal, hepatic, and thyroid function tests also returned normal results. Cranial and orbital MRI with contrast showed no noticeable structural abnormalities (**Fig. 2B**).

To explore potential causes, the patient was referred to a neurologist for further evaluation. The neurologist found no signs of aphasia or dysarthria, and her language interpretation was normal. The patient’s limb strength, deep tendon reflexes, and flexor plantar responses were also within normal limits. The neurologist ruled out meningitis or other infectious causes due to the absence of signs of meningeal irritation. However, a cerebrospinal fluid (CSF) examination revealed a low white cell count (<1/mm^3^), normal protein levels, and normal glucose content, ruling out infections. Serology tests for various viruses, including Rubella, Borrelia, and herpes simplex virus, returned negative results.

Despite these normal findings, further investigations were required to identify the cause of the ophthalmoplegia and ptosis. The neurologist ordered a serum anti-ganglioside antibody panel, which revealed high levels of anti-GQ1b IgG (+++), anti-GQ1b IgM (++), and anti-GD1a IgM (++), strongly suggesting Miller-Fisher syndrome (MFS), a variant of Guillain-Barré syndrome (GBS). These results explained the patient’s symptoms and prompted treatment with intravenous immunoglobulins (IVIG), given at a dosage of 2 g/kg for five days.

Following the treatment with IVIG, the patient showed significant improvement. Diplopia, ptosis, and eye motility improved within four weeks. At the fifth month of follow-up, the symptoms of diplopia and signs of ptosis were completely resolved, and eye motility returned to normal.

### Case 4: Optic Neuritis Leading to Diagnosis of NMOSD

An 8-year-old girl, the second child from a non-consanguineous marriage, presented to the Ophthalmology outpatient department with a complaint of sudden vision loss in both eyes, with the left eye being more severely affected. The vision loss had begun abruptly 10 days prior, reaching its peak within 1 to 2 days. The patient also experienced a sensory seizure of the right parietal lobe, which manifested as a vague sensation in the left upper limb, followed by weakness and loss of consciousness. Her distant visual acuity was found to be 6/60 in the right eye, improving to 6/24 with a pinhole, while the left eye had a best-corrected visual acuity (BCVA) of hand movements close to the face. Further ophthalmological examination revealed a RAPD grade 4 in the left eye. Anterior segment examination of both eyes appeared normal. Still, posterior segment examination revealed temporal pallor in the right eye and blurring of the disc margins in the left eye, suggesting optic nerve involvement. These findings indicated significant visual impairment and raised concern for a more serious underlying condition.

**Fig. 3:**
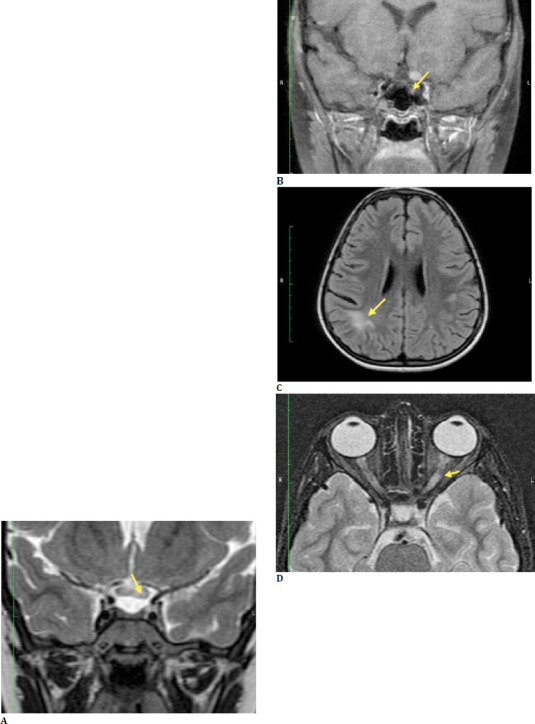
**A**. T2 coronal image of an eight-year-old child shows edema in the left optic nerve extending to the optic chiasma; **B**. STIR axial image shows thickened optic nerve; **C**. T2 FLAIR axial image shows juxtacortical white matter lesions in the right parietal lobe; **D**. T1 post-contrast coronal image shows enhancement of the left optic nerve. No enhancing lesion is observed in the brain parenchyma. However, two subtle focal enhancing lesions are observed in the cervical and dorsal cord (images not shown)

Given the sudden vision loss and abnormal neurological findings, an MRI of the brain and spine was ordered to investigate the cause further. The MRI revealed hyperintensities in the left optic nerve, optic chiasma, and bilateral optic tracts, along with thickening of the left optic nerve (**Fig. 3A, B**). Further hyper-intensities were noted in the white matter of the frontal, parietal, and temporal lobes, with some lesions in the juxta-cortical region of the right parietal and medial temporal lobes (**Fig. 3C**). Additionally, post-contrast images revealed enhancement along the left optic nerve and optic chiasma, consistent with inflammation (**Fig. 3D**). These findings suggested a demyelinating disorder, with NMOSD as a likely diagnosis.

The patient was started on intravenous methylprednisolone at 1 gram per day for three days, followed by oral prednisolone at a dose of 30 mg/kg/day. She was then transferred to a pediatric neurologist. There, the diagnosis of NMOSD was confirmed with a positive test for anti-myelin oligodendrocyte glycoprotein (anti-MOG) antibodies. Additional CSF analysis revealed elevated protein and glucose levels, indicative of a demyelinating process.

After treatment with steroids, the patient’s BCVA improved to 6/9 in the right eye and 6/24 in the left eye, with no evidence of optic nerve edema. Continued follow-up showed a gradual resolution of demyelination in the optic nerve, tracts, and spine. The patient entered a remittance phase, with no further signs of neurological or visual deficits.

## Discussion

The presented cases emphasize the crucial role of detailed ophthalmological evaluation in identifying and diagnosing a range of neurological conditions. The eye can be the first organ to manifest subtle signs of systemic illnesses.

In the first case, the observation of a critical vascular anomaly through an eye examination underscored the importance of thorough ocular examination in identifying potentially life-threatening conditions. Such aneurysms can remain asymptomatic if they are small or may progress to cause headaches and cranial nerve palsies due to the mass effect of the aneurysm on the anterior optic pathway [3,4]. Blood supply to this pathway can also be compromised by compression on the ophthalmic artery [3,4].

The management of unruptured supraclinoid carotid aneurysms involves a variety of approaches [23,24]. While MRI is frequently used to diagnose these aneurysms, digital subtraction angiography is considered the gold standard for confirmation and planning for surgical or endovascular treatment [23,24]. Surgical clipping and endovascular coiling are two commonly used treatment methods that effectively prevent rupture. At the same time, conservative management is typically reserved for asymptomatic aneurysms measuring less than 10 mm, followed by regular imaging follow-up [23,24].

The second case further demonstrated the comprehensive role of ophthalmological evaluation in patient care, where the discovery of a third nerve palsy led to a deeper investigation, ultimately revealing metastatic multiple myeloma. This aligned with existing literature indicating that ocular manifestations can be early indicators of systemic malignancies [25]. Intracranial plasmacytomas are rare plasma cell neoplasms, constituting less than 1% of all intracranial tumors [25,26]. The proximity of these tumors to cranial nerves, especially the abducens nerve, can lead to additional cranial nerve palsies and neurological symptoms [25,26]. Treatment options for plasmacytomas include surgery, radiation therapy, and chemotherapy, with the choice depending on the tumor size and location [25,26]. This case illustrated the importance of thorough ophthalmological evaluations in diagnosing systemic malignancies and informing appropriate treatment plans.

The third case emphasized the importance of interdisciplinary collaboration in diagnosing autoimmune neurological syndromes. The identification of MFS through collaborative efforts between ophthalmologists and neurologists resulted in prompt treatment with intravenous immunoglobulins (IVIG), leading to significant improvement in the patient’s condition. This case highlighted the importance of ophthalmologists recognizing ocular signs that might indicate broader autoimmune conditions, aligning with the literature that supports the potential for ocular manifestations in autoimmune conditions [27].

Lastly, the final case illustrated the role of ocular evaluation in diagnosing neuromyelitis optica spectrum disorder (NMOSD). The detection of optic neuritis and related visual impairments led to a series of investigations confirming the diagnosis, eventually leading to successful treatment with steroids. Due to the rarity of this disorder, establishing consensus guidelines for management is challenging [28,29]. Treatment for NMOSD involves high-dose corticosteroids and plasmapheresis during acute attacks, aiming to reduce inflammation and neurological damage [28,29]. Long-term immunosuppressive therapies, including Food and Drug Administration (FDA)-approved drugs such as eculizumab, inebilizumab, and satralizumab, are crucial for preventing relapses [28,29]. Prognosis varies widely; some patients experience severe, frequent attacks leading to significant disability, particularly in vision and mobility [28-29]. However, early diagnosis and prompt, effective treatment can improve outcomes, reducing the likelihood of severe impairment and mortality. Despite these advances, many patients still face considerable challenges, emphasizing the need for ongoing medical support and monitoring to enhance quality of life.

These cases underscore the crucial role of a detailed ocular examination in facilitating early diagnosis and achieving successful outcomes. They emphasize the importance of early detection and interdisciplinary collaboration, demonstrating that the prompt identification of ocular signs can lead to life-saving interventions. To our knowledge, no such case series has been reported previously. Future research should focus on larger cohorts to validate these findings and further explore the mechanisms linking ocular health to systemic diseases.

## Conclusion

The presented cases exemplify the early ocular signs of life-threatening neurological conditions. These cases underscore the importance of comprehensive ophthalmological examinations in identifying a wide range of medical pathologies.
